# Conceptualising compensation in neurodevelopmental disorders: Reflections from autism spectrum disorder

**DOI:** 10.1016/j.neubiorev.2017.06.005

**Published:** 2017-09

**Authors:** Lucy Anne Livingston, Francesca Happé

**Affiliations:** MRC Social, Genetic and Developmental Psychiatry Centre, Institute of Psychiatry, Psychology and Neuroscience, King’s College London, De Crespigny Park, London, SE5 8AF, UK

**Keywords:** Autism Spectrum Disorder, Neurodevelopmental disorders, Compensation, Compensatory mechanisms, Adaptation, Camouflaging, Cognitive phenotype, Behavioural phenotype, Remediation, Theory of mind, Executive function, Good outcome, Under-diagnosis, Late diagnosis, Female presentation, Unaffected siblings

## Abstract

Within research into neurodevelopmental disorders, little is known about the mechanisms underpinning changes in symptom severity across development. When the behavioural presentation of a condition improves/symptoms lessen, this may be because core underlying atypicalities in cognition/neural function have ameliorated. An alternative possibility is ‘compensation’; that the behavioural presentation *appears* improved, despite persisting deficits at cognitive and/or neurobiological levels. There is, however, currently no agreed technical definition of compensation or its behavioural, cognitive and neural characteristics. Furthermore, its workings in neurodevelopmental disorders have not been studied directly. Here, we review current evidence for compensation in neurodevelopmental disorders, using Autism Spectrum Disorder as an example, in order to move towards a better conceptualisation of the construct. We propose a transdiagnostic framework, where compensation represents the processes responsible for an observed mismatch between behaviour and underlying cognition in a neurodevelopmental disorder, at any point in development. Further, we explore potential cognitive and neural mechanisms driving compensation and discuss the broader relevance of the concept within research and clinical settings.

## Highlights

• Compensation may underpin improvements in symptoms in neurodevelopmental disorders.
• The construct of compensation is poorly understood and has no agreed definition.
• We derive a working definition and review evidence for compensation (e.g., in ASD).
• We propose a preliminary transdiagnostic framework of compensation.
• We discuss potential neurocognitive mechanisms and research/clinical implications.

## 1 Introduction

Much research into neurodevelopmental disorders has focused on uncovering the cognitive and neurobiologial atypicalities that underlie the defining behavioural symptoms of the condition in question. In the case of Autism Spectrum Disorder (ASD), numerous cognitive theories (e.g., deficient ‘theory of mind’, ‘weak central coherence’; see Frith, 2012) have been proposed to account for the social and non-social symptoms that are characteristic of the condition. And yet, across neurodevelopmental disorders and pertinent in the case of ASD, there is great heterogeneity in the degree to which symptoms lessen, persist or even worsen across the lifetime. The mechanisms by which the behavioural presentation of a condition might alter across development remain largely elusive. For example, in the case of ASD, there currently exists no empirically-grounded explanation pertaining to why some autistic children no longer fulfil diagnostic criteria by adulthood and likewise, why some autistic individuals do not present with clinically impairing symptoms *until* adulthood.

It is tempting to assume that any significant improvement in the behavioural presentation of a neurodevelopmental condition has come about due to some alteration or alleviation of atypical underlying cognition and/or neural function. In an authoritative opinion piece on the last 25 years of research into ASD and developmental dyslexia, Uta Frith highlighted that “it is still not clear what causes these changes and wide variations [in behavioural symptoms]…but compensation makes it possible to disguise persisting problems” (2013, p. 670). This begs the question; what is compensation and how can we measure it? Within the field of neurodevelopmental disorders, the phenomenon remains relatively abstract and ill-defined, such that numerous, potentially overlapping terminologies have been used in the literature (e.g., in ASD, camouflaging/masking, Lai et al., 2016; compensatory learning, Frith, 1991; adaptation, Johnson et al., 2015b), but the construct has never been directly studied in its own right. Nevertheless, the importance of understanding compensation in neurodevelopmental phenotypes is clear. First, given increasing interest in studying previously conceived ‘childhood’ disorders, such as ASD and Attention Deficit Hyperactivity Disorder (ADHD), across the whole lifespan, including how disorder presentation might change over time (for ASD, see Georgiades et al., 2017), developmentally-relevant mechanisms for improvement, including compensation, should be an important focus of research. Second, compensation could be a useful way to unpick some of the heterogeneity amongst neurodevelopmental disorders, which is frequently proposed to be one of the greatest challenges to understanding these conditions (Thapar et al., 2017). Finally, studying the mechanisms underlying compensation could be fundamental to informing early intervention research, whose principle aim is to improve long-term prognosis. And yet, in order to directly investigate compensation, we must have a reasonable definition to guide our measurements and derive testable hypotheses.

In this paper, we aim to create a working definition of compensation relevant to neurodevelopmental disorders and use this definition to i) review evidence for compensation, garnering examples from ASD, ii) propose a preliminary framework of the workings of compensation, iii) explore its potential cognitive and neural underpinnings, and finally, iv) discuss the research and clinical implications of studying neurodevelopmental disorders within this compensation framework.

## 2 Defining and measuring compensation in neurodevelopmental disorders

### 2.1 Compensation in the psychological literature

Within psychological research in general, the term ‘compensation’ has been widely used. In instances where participants, who are expected to be limited in a particular set of resources (be this due to a psychiatric condition, old age, or an experimental manipulation amongst healthy participants), perform better than expected on a psychological task, the possibility that they have in some way compensated, is often speculated upon by authors. This compensatory hypothesis is generally backed up by evidence showing that ‘compensated’ participants have achieved this ‘typical’ performance with the recruitment of additional resources, be these neurobiological, cognitive, or genetic. For example, in the literature on aging, researchers have used the term to describe how older adults can demonstrate atypical activation (enhanced or decreased) of task-relevant brain areas or activation of additional regions not typically recruited by younger adults, in order to perform a task just as well as their younger counterparts (see Grady, 2012). Equally, within research into neuropsychological patients, the term ‘compensation’ refers to the brain’s ability to rely on alternative neural routes after typical routes have been compromised by brain damage, in order for patients to make improvements in behaviour/cognitive abilities (e.g., Price and Friston, 2002). The term ‘partial compensation’ is used to describe how an attempt to counteract limited resources may not always be efficient enough to support wholly ‘typical’ behaviour or cognitive task performance.

Despite frequent use of the term ‘compensation’, there is no technical or universal definition. The precise interpretation of its meaning or the meaning of ‘compensatory brain activity’ is specific to the particular task and participant population in question. The literature also suggests that the process of compensation could actually exist and operate at multiple levels, from molecular and/or genetic pathways (for instance, synaptic plasticity in order to counteract atypical connectivity; e.g., in ASD, Bourgeron, 2015) to broader cognitive systems and behaviour (for instance, atypical neural functioning to support typical cognitive task performance; e.g., in ASD, White et al., 2014). In this paper, due to the complexity and novelty of the phenomenon, we will focus on conceptualising compensation across levels of behaviour, cognition and whole neural networks only, solely within the scope of neurodevelopmental disorders.

### 2.2 Compensation in the literature on neurodevelopmental disorders

With no agreed definition, it is unsurprising that compensation in neurodevelopmental disorders has received little empirical attention. There is, to our knowledge, only one review paper on the topic (Ullman and Pullman, 2015), which explores the specific compensatory function of the declarative memory system in five neurodevelopmental disorders, including ASD. Crucially, the authors’ review relies on a definition of compensation that is reminiscent of that described in the aging and neuropsychological literature; that compensation reflects how an intact neurocognitive process/system might take over, or *compensate for*, the functioning of a defective process/system in order to maintain typical behaviour and/or cognitive task performance. Indeed, Ullman and Pullman (2015) suggest that in ASD, where socio-cognitive functioning is compromised, intact declarative memory ability may scaffold social behaviour; for example, the ability to recall previously learned social rules may replace intuitive understanding of social cues, thereby contributing to an appropriate social response.

There is, however, good reason to question whether a definition derived from the study of individuals who have *acquired* their deficits (e.g., brain-damaged individuals/aging adults), necessarily extends to neurodevelopmental populations (Johnson, 2017, Thomas and Karmiloff-Smith, 2002). For example, Johnson (2017) has highlighted how focal brain damage during the pre/perinatal period may be compensated for by early reallocation of function to intact brain regions, but that in the case of conditions such as ASD, where more wide-spread early brain disturbance is observed (or postulated, e.g., general synapse dysfunction), an alternative explanation of compensation may be required. Additionally, brain injury in healthy adults may trigger a host of compensatory processes (e.g., enhanced connectivity from damaged to frontal regions; Sharp et al., 2014) that are not necessarily comparable to cases where a cascade of atypical neural function has existed from very early in development.

In our endeavor to find a definition of compensation drawn from observations in neurodevelopmental phenotypes, we take inspiration from research into a developmental condition that has a relatively circumscribed cognitive deficit and has received some preliminary discussion with regards to compensation; namely developmental dyslexia.

### 2.3 Lessons from developmental dyslexia

Developmental dyslexia is characterised by a specific impairment in reading, not otherwise accounted for by intellectual or visual abilities (American Psychiatric Association, 2013). The condition is proposed to be underpinned by a core deficit in phonological processing (Snowling, 2014), which contributes to an array of behavioural symptoms amongst dyslexics (e.g., spelling errors and slow reading and word recognition; Thambirajah, 2010). Critically, although the majority of children with dyslexia experience these difficulties persistently (Hatcher et al., 2002, Maughan et al., 2009), a subset of individuals, referred to as ‘compensated dyslexics’ (Lefly and Pennington, 1991), eventually establish typical reading skills by the time they enter adulthood (Callens et al., 2012, Gallagher et al., 1996).

In principle, there are at least three possible ways in which dyslexic individuals’ primary symptoms could lessen. First, the phonological processing deficit at the cognitive level may genuinely remit, thus supporting good reading ability. Second, phonological processing may be delayed rather than deficient in these children, so that there is eventually developmental ‘catch up’. Third, good reading ability may be facilitated by alternative neurocognitive pathways that are independent of phonological routes. On inspection of the literature, the first two possibilities do not appear to hold up empirically. Amongst highly ‘compensated’ dyslexics, who do not exhibit measurable spelling or reading difficulties (e.g., those in higher education), significant phonological processing deficits are revealed when tapped with sensitive enough cognitive probes, such as rapid picture naming (Gallagher et al., 1996, Ingvar et al., 2002, Parrila et al., 2007, Swanson, 2012). Further, these individuals’ reading abilities are not necessarily comparable to those of typically developing individuals under certain contexts; for example, their reading speeds are significantly slower than typically developing individuals’ when put under a time constraint (Parrila et al., 2007), or when exposed to substantial background noise (Varnet et al., 2016).

A more plausible explanation is that ‘typical’ reading ability is achieved via one or more atypical routes that bypass phonological processing. These alternative processes may be sufficient in certain contexts, but ultimately, are not as fine-tuned as phonologically-based routes to reading. This is in line with empirical findings and self-reports of alternative cognitive strategies amongst dyslexics; for example, fitting the orthographic forms of unfamiliar words to familiar ones where spelling of the two is similar (Cavalli et al., 2016, Parrila et al., 2007) and relying upon visual imagery (Bacon and Handley, 2014) and the semantic context of words (Corkett and Parrila, 2008, Pugh et al., 2001). This notion of non-phonological routes to ‘typical’ reading is further corroborated by evidence at the neural level, such as findings of neural signatures that are unique to ‘compensated’ (versus ‘uncompensated’) dyslexics when performing phonological tasks (Ingvar et al., 2002, Shaywitz et al., 2003). Amongst ‘compensated’ dyslexics, neuroimaging findings generally support hypoactivation of areas implicated in phonological function, e.g., left parieto- and occito-temporal areas (Cao et al., 2008, Hoeft et al., 2007), and hyperactivation of additionally recruited areas, e.g., right inferior frontal gyrus (Hoeft et al., 2011, Shaywitz et al., 2004). Greater functional connectivity between posterior visual regions has also been reported (Koyama et al., 2013), perhaps reflecting the potential compensatory role of visual strategies (for an exhaustive review of compensatory neural pathways, see Pammer, 2014).

### 2.4 Working definition of compensation

Developmental dyslexia is a particularly useful model from which to guide a working definition of compensation as it is one of the few neurodevelopmental conditions in which the underlying cognitive deficit can be measured fairly accurately (even in so-called ‘compensated’ adults), thereby allowing for distinct measurements of behaviour and cognition. This is important as the developmental dyslexia literature suggests that compensation signifies some discrepancy between the *perceived* ability of an individual, exhibited in their behaviour (i.e., degree of observable symptoms), and *actual* ability, exhibited in underlying cognitive and/or neural function. Accordingly, our working definition of compensation is as follows: *the processes contributing to improved behavioural presentation of a neurodevelopmental disorder, despite persisting core deficit(s) at cognitive and/or neurobiological levels*. This definition is intentionally general (i.e. transdiagnostic), whilst still making qualitative predictions about what lies at behavioural, cognitive and/or neural levels.

The principal proposal of this definition is that compensation contributes to a mismatch between behaviour and the relevant underpinning cognition, whereby a ‘compensated’ individual’s behavioural presentation is better than would otherwise be expected based on their underlying cognitive profile. The mechanism of compensation, therefore, contrasts with instances where behaviour and cognition remain matched, for example, where behaviour improves due to a real lessening of the core underlying deficit (i.e., genuine remission). A special case of the latter would be delayed maturation (see Table 1), whereby a cognitive ability is delayed in ‘coming online’. We suggest that in cases of delayed maturation, the underlying ability eventually becomes neurotypical, although its late emergence may leave ‘scars’ because a critical window has been missed in development; for example, connections with typical sensory inputs may not wire in the usual way. However, in contrast to compensation, we would expect those individuals whose cognitive ability matured late to show improvement not only in behaviour but also in their underlying cognitive profile, albeit atypically. Critically, we predict that a ‘compensated’ person, i.e., an individual that *appears* to be doing well, is not necessarily exhibiting a milder form of their condition. Instead, severe and persistent underlying deficits may exist, but these are masked by the presence of a substantial amount of compensation (Frith, 2004).

**Table 1:** Hypothetical distinctions between different mechanisms that could promote improved outcome; Compensation (shallow), Compensation (deep), Genuine remediation, Delayed maturation. Social skills at the behavioural level and theory of mind (ToM) at the cognitive level in ASD are used as an example here, but could be substituted with another behaviour, underlying cognitive ability and neurodevelopmental disorder. These predictions require empirical testing.

	Compensation (shallow)	Compensation (deep)	Genuine remediation	Delayed maturation
Behavioural	Good social skills in structured contexts (e.g., in the ADOS assessment), but these do not hold up in everyday situations where social cues are ambiguous and fast-paced.	Good social skills in structured contexts and everyday situations.	Good social skills in structured contexts and everyday situations.	Good social skills in structured contexts, but these may not hold up in complex social situations, where, e.g., multiple sources of information must be integrated.

Cognitive	Clear ToM deficit when measured with sensitive ToM tasks (e.g., implicit ToM tasks). Individuals may be able to solve explicit ToM tasks through logical reasoning.	No clear ToM deficit as compensation has extended to the cognitive level. However, good ToM task performance might come at a cost to time (e.g., good accuracy but slow response times).	No ToM deficit on implicit or explicit ToM tasks. ToM deficit has genuinely resolved.	No clear ToM deficit but ToM may have matured too late to have dynamic input into the system. Individuals may be able to attribute mental states, but not as fast and without integrating multiple cues.

Neural	ASD-associated brain atypicalities persist (e.g., atypical patterns of brain activation during ToM tasks).	Atypical neural route in order to support good ToM task performance (e.g., extra neural ‘effort’ or recruitment of additional brain areas).	Unknown. Potentially a combination of typical neural markers and traces of early ASD markers.	Unknown. Could be lasting functional atypicalities (e.g., connectivity to other brain regions may be different) if critical windows for neural integration of different functions are missed.

Genetic	Substantial genetic load for ASD, but also genetic propensity for good general-purpose cognitive skills (e.g., IQ, executive function).	Substantial genetic load for ASD, but also genetic propensity for good general-purpose cognitive skills (e.g., IQ, executive function).	Reduced genetic load for ASD compared to compensators.	Reduced genetic load for ASD compared to compensators.

Other Characteristics	Effortful, absorbs central domain-general resources; hence, breaks down under stress/anxiety and may come at a cost to mental health.	Potentially some cognitive/neurobiological marker for ASD in earlier development. Mental health may not be negatively impacted.	No more effortful than for neurotypicals; for instance, perform no differently to neurotypicals when resources are limited (e.g., during dual task challenges).	Longitudinal studies may demonstrate neurotypical order of skill acquisition, but with substantial delay.

**Fig. 1:**
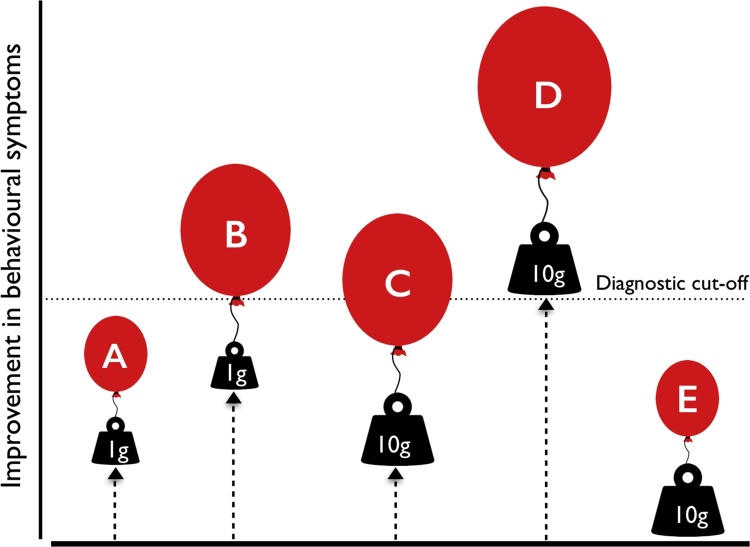
Visual representation of compensation, where A–E represent five hypothetical individuals with a given neurodevelopmental disorder. The black weights represent the extent of core cognitive deficit associated with that disorder (e.g., in ASD, theory of mind deficit). Larger weights (10g) versus smaller weights (1g) represent greater severity of the cognitive deficit. The balloons represent the extent of compensation that has taken place. Larger versus smaller balloons represent a greater degree of compensation. The vertical dashed arrows represent the extent of improvement in the behavioural presentation of the disorder/lessening of symptoms. The higher the vertical dashed arrow extends upwards, the greater the improvement in the behavioural presentation of the disorder. The horizontal dashed line represents a hypothetical diagnostic cut-off, below which symptoms are sufficiently impairing to meet criteria for a diagnosis and above which, diagnostic criteria are not met.

In conceiving compensation as the processes contributing to a behaviour-cognition mismatch, the critical obstacle to investigating the phenomenon within neurodevelopmental disorders, which are for the most part diagnosed by behaviour alone, becomes strikingly clear. The behavioural presentation of a disorder arises from the combination (and possible interaction) of both core cognitive deficits and the compensatory processes that have taken place to ameliorate the expression of these deficits, at any point in development. Fig. 1 depicts theoretically what the implications of this might be, by presenting five hypothetical individuals (A–E) within a neurodevelopmental population, each with their own degree of i) core cognitive deficit, ii) behavioural symptoms and iii) compensation.

First, the extent of the cognitive deficit is not systematically related to the degree of symptom severity, i.e., there is a clear mismatch between behaviour and cognition. Second, two individuals can appear behaviourally ‘identical’ despite very different doses of deficit and compensation (A and C). Equally, two individuals can have equally severe deficits but appear substantially different in behaviour, due to varying amounts of compensation (A and B). Third, an individual’s symptoms may appear to have lessened, perhaps to the extent that they no longer fulfil diagnostic criteria, despite a substantial core cognitive deficit (D). Fourth, those with a double hit of a severe deficit and also poor ability/propensity to compensate will appear the most behaviourally severe (E).

It should be noted that the model in Fig. 1 assumes that the extent of compensation taking place is independent of the degree of core cognitive deficit, thereby allowing for all possible combinations of the two. In some cases, however, the ability to compensate may be limited by the cognitive deficit itself; for example, insight into one's own difficulties might be necessary to trigger compensation. The figure also omits, for simplicity, possible additional adverse risk factors that might contribute to overall disorder presentation (e.g., low socio-economic status, ethnic status, home chaos). Whilst these could be seen as factors that simply strip away compensatory resources, adverse environments may also directly influence disorder presentation (i.e., interact with the expression of underlying deficits in behaviour), for example, by increasing vulnerability to further stress/adversity or substantiating resistance to future adversity (Rutter, 2006, Rutter, 2012).

### 2.5 Measuring compensation

According to our working definition, a purely behavioural tool would be insufficient for measuring compensation, as the very definition reflects the disparity between behavioural and cognitive levels. For the purpose of research, our measurement tool must be able to disentangle the contributions of core cognitive deficit and compensation to the behavioural presentation of the disorder. This will, as demonstrated by the literature on developmental dyslexia, require highly sensitive and, ideally, process-pure cognitive probes that are not dependent on the symptoms themselves. Further, our cognitive tasks should be able to detect subtle difficulties in even the most intellectually able of individuals. Indeed, well-compensated, highly able individuals may demonstrate reasonable performance on cognitive tasks aiming to tap the deficit in question, but this might come at the cost of time and/or other cognitive resources (Frith, 2013). Future research into compensation might therefore benefit from more fine-grained data collection, such as the speed of task performance or the effects of dual task interference.

## 3 Evidence for compensation in ASD

Autism Spectrum Disorder (ASD) is a set of neurodevelopmental conditions characterised by social and communication impairments and repetitive and restricted behaviours and interests (American Psychiatric Association, 2013). One prominent explanation of the social symptoms in ASD posits a core cognitive deficit in theory of mind (Baron-Cohen et al., 2000), which is the propensity to infer spontaneously what others are thinking in order to predict and explain their behaviour (Happé, 2015). As there is evidence to suggest that cognitive and neural atypicalities associated with ASD may contribute differentially to the social and non-social symptoms (Happé et al., 2006), we will here focus on observations of compensation in the social domain only; where individuals’ social symptoms appear improved, despite persistence in an underlying theory of mind deficit.

We should note that theory of mind is just one example of a socio-cognitive ability that may be atypical in ASD (see Happé et al., 2017), which we use here for the purpose of simplifying our model. This could in theory be substituted for other socio-cognitive probes or biological markers that underpin social symptoms in ASD. Traditionally, theory of mind tasks require participants to attribute mental states to others (e.g., that someone holds a false belief about the world) in verbal vignettes (e.g., Strange Stories; Happé, 1994), pictures/cartoons/animated shapes (e.g., Frith-Happé Animations; Abell et al., 2000), or naturalistic videos (e.g., Strange Stories Films Task; Murray et al., 2017). Explicit theory of mind tasks usually require participants to answer questions about what the characters think, while implicit tasks measure, e.g., anticipatory eye movements during a false belief scenario, without a direct mental state question. We have identified four themes in ASD where we suggest compensation is relevant: i) good outcome, ii) at-risk individuals transitioning away from the autism phenotype, iii) late diagnosis, and iv) female autism presentation.

### 3.1 Good outcome

Individuals diagnosed with ASD in childhood who go on to achieve ‘good outcome’ have been described as showing remarkable compensation (Frith, 2013). These are, according to a body of longitudinal and retrospective studies, the subset of individuals on the autism spectrum who, by adulthood, display markedly reduced autistic symptoms (Farley et al., 2009, Helles et al., 2015, Lord et al., 2015, Magiati et al., 2014, Seltzer et al., 2004) and achieve a degree of success in terms of educational/occupational status, independence, and social relationships (Billstedt et al., 2005, Eaves and Ho, 2008, Farley et al., 2009). We do note, however, that the majority of studies into adult outcome thus far rely heavily on definitions of outcome imposed by neurotypicals to neurotypical standards, and may not necessarily reflect the best outcomes as perceived by autistic individuals themselves. These studies to date consider ‘good outcome’ to have occurred, when autistic individuals appear, at least on the surface, to be experiencing a less impairing version of the condition. A similar pattern is seen amongst other neurodevelopmental disorders, including developmental dyslexia, as discussed earlier, and ADHD. For example, a third of children with ADHD no longer exhibit clinically impairing symptoms by adulthood (Faraone et al., 2006). Crucially, if this ‘good outcome’ has arisen despite persistence in underlying core cognitive deficits, rather than because these deficits have genuinely resolved, these individuals represent a subgroup that fit our aforementioned definition of compensation.

There is good reason to suspect that autistic adults with ‘good outcome’, who typically show good intellectual and language abilities, continue to have characteristically autistic cognitive profiles, despite displaying “[social] behaviour that more and more shades into normality” (Frith, 1991, p. 31). Socially adapted behaviour might be achieved via overt and conscious strategies, despite core socio-cognitive abilities, such as theory of mind, remaining impaired. For example, an individual may be taught to make eye contact without necessarily being able to extract mental state information from that eye contact. Further, there is evidence to suggest that logical ‘hacking’ and intact verbal abilities might facilitate success on explicit theory of mind tasks that directly ask participants to reason about mental states, in both children (e.g., Cantio et al., 2016, Happé, 1995, Peterson et al., 2007, Scheeren et al., 2013) and adults (Lever and Geurts, 2016a, Senju et al., 2009). Such strategies, however, cannot support performance on more sensitive, implicit measures of theory of mind (e.g., anticipatory eye gaze based on attribution of a false belief), in which persistent difficulties are revealed (Senju et al., 2009, Schneider et al., 2013). This notion of a fundamental and enduring difficulty in understanding others’ minds is further highlighted by the fact that in situations where learned strategies may not be sufficient to fully compensate, such as novel, fast-paced everyday social interactions, even ‘good outcome’ individuals report difficulty ‘keeping up’.

Evidence for a substantial mismatch between observable social behaviour and underlying theory of mind, as described above, amongst autistic individuals who otherwise appear socially adapted casts doubt on claims that some autistic people may genuinely transition off the autism spectrum; for instance, in research describing children who reach ‘optimal outcome’ after early behavioural intervention (Fein et al., 2013, Helt et al., 2008). These studies report on individuals who had a reliable diagnosis of ASD in childhood yet by young adulthood are indistinguishable from their typically developing peers, even in the absence of any additional support. The extent to which these individuals are showing compensation in the face of continuing underlying difficulties, rather than a genuine ‘remission’ of ASD, is yet to be explored. Crucially, the underlying socio-cognitive profile of these individuals has not been empirically investigated. However, reports of continued social awkwardness and reduced quality of friendships (Orinstein et al., 2015) suggest that these individuals continue to experience significant social difficulties. This points to a broader issue within research into outcome in ASD, which has predominantly relied upon behavioural measures. If we are to gain a better understanding of compensation amongst these highly able individuals, a closer, more in-depth look at the discrepancy between behavioural and cognitive outcomes will be required.

### 3.2 At-risk individuals transitioning away from the autism phenotype

The prospective study of infants at high genetic and/or environmental risk for ASD (those with an older sibling with an ASD diagnosis) has demonstrated that a substantial proportion (20%) of these individuals will also go on to follow an autism trajectory (Elsabbagh and Johnson, 2010). Of particular interest to the notion of compensation, are those at-risk siblings who, despite displaying some early ‘red flags’ for autism at 12 months of age (for review, see Jones et al., 2014), are indistinguishable from typically developing individuals in terms of social and communicative behaviour by 3 years of age. For example, Macari et al. (2012) found this trajectory amongst 9% of their sample of at-risk siblings. To the extent that these early behavioural markers reliably predict later ASD diagnosis, rather than broader developmental issues (e.g., ADHD, cognitive impairment), these individuals might represent some form of early, unconscious compensation, which buffers against the expression of ASD in behaviour.

Johnson et al. (2015b) have proposed that, in the face of substantial risk for ASD in early development, a series of adaptive/compensatory neural processes might take place, thereby reducing the severity of autistic symptoms manifested in behaviour later on. This may be to the extent that certain individuals bypass an autism diagnosis altogether. Equally, the most severe symptom presentations might signal a very limited capacity for this early neural compensation, which Johnson (2012) suggests is rooted in the availability of prefrontal cortex (PFC) based executive functions. This standpoint poses an intriguing theoretical possibility concerning the broader autism phenotype (BAP), which is the sub-clinical expression of autistic traits, often seen in relatives of those with ASD (Sucksmith et al., 2011). Traditionally, BAP has been explained in terms of unaffected relatives having a milder ‘hit’ for ASD (e.g., milder theory of mind deficit) compared to their affected relative, which keeps them below the diagnostic threshold. However, reframing this in light of compensation, opens up the possibility that in some cases, unaffected relatives may not necessarily possess a milder cognitive burden for ASD *per se*, but instead, possess a greater capacity to compensate for this cognitive burden (Skuse, 2007), resulting in an apparently milder behavioural presentation. This does not exclude the possibility, which is indeed the prevailing view, that many relatives express a milder phenotype due to genuinely milder underlying autistic difficulties.

In order to assess the extent to which unaffected siblings might in some cases be exceptional at compensating, one could investigate a cognitive probe or biological marker shared amongst individuals with ASD and their siblings (i.e., an endophenotype), which signals some kind of aetiological load or ‘hit’ for ASD (here we have focused on theory of mind impairment, but other examples could include attentional abnormalities or EEG markers; see Jones et al., 2014). Compensation could be inferred when so called ‘unaffected’ siblings are demonstrating a typical behavioural profile, in spite of the presence of a cognitive deficit or biological marker characteristic of those with diagnosed ASD. By contrast, unaffected siblings who appear typical in terms of the cognitive characteristic/biological marker of interest, would likely not be compensating, but rather, showing a typical behavioural profile due to a genuine lack of ‘hit’ for ASD. Methods that aim to quantify aetiological load for ASD within the general population could help clarify this distinction between unaffected siblings who have a lesser ‘hit’ for ASD versus those who are compensating in the face of a substiantal ‘hit’; for example, siblings that are compensating should demonstrate high common genetic risk for ASD. This could, for example, be estimated using polygenic risk scores, which are based on single nucleotide polymorphisms associated with the disorder in question. Furthermore, it will be useful to determine if siblings can compensate for rare genetic risk (e.g., *de novo* mutations) by investigating the genetic patterns of all affected/unaffected members of a family, alongside cognitive/biological markers for ASD.

In the case of theory of mind, highly sensitive tasks will be required to reveal subtle difficulties with mentalising in unaffected siblings. Currently, there are only a few theory of mind tasks that are able to capture individual differences in typically developing individuals (e.g., Movie for the Assessment of Social Cognition, Dziobek et al., 2006; Strange Stories Film Task, Murray et al., 2017; Adult-Theory of Mind, Brewer et al., 2017). Continued longitudinal investigation of siblings will also be required to clarify whether those who demonstrate early autistic-like features that eventually disappear are genuinely compensating for an underlying liability for ASD specifically, which might be revealed with a cognitive or biological probe, or whether these ‘red flags’ in fact signal reversible social atypicalities, for example, due to developmental delay.

### 3.3 Late diagnosis

Since the earliest reported cases over 70 years ago (Asperger, 1944 [translated in Frith, 1991]; Kanner, 1943), autism has been conceptualised as a condition with childhood-onset. However, many individuals come for an ASD diagnosis for the first time in adulthood (Geurts and Jansen, 2012, Happé and Charlton, 2012, Lai and Baron-Cohen, 2015). This is thought in part to mirror increasing public awareness of ASD and a substantial widening of diagnostic criteria (Hansen et al., 2015, Rutter, 2005). Additionally, the latest addition of the *Diagnostic and Statistical Manual of Mental Disorders*, DSM-5 (American Psychiatric Association, 2013), has for the first time recognised that autistic symptoms “may not become fully manifest until social demands exceed limited capacities” (p. 50). Hence, the underlying assumption regarding first diagnosis in adulthood, is that core autistic difficulties have always existed for these individuals, but the ability to compensate in some way has supported a relatively ‘neurotypical’ presentation, at least up until adulthood, where social demands have exceeded compensatory ability. Alternatively, these individuals diagnosed in adulthood may have exhibited some autistic behaviours earlier in life, but these symptoms were not sufficiently impairing for a diagnosis. For example, certain individuals may construct a niche for themselves that lessens the burden of their social difficulties (e.g., having a partner to act as their ‘social brain’, an employment environment that is low in social demands) and only when this niche is disturbed do their autistic behaviours become impairing enough to seek and warrant a diagnosis (see Section 4.2).

Demographically, this group of late diagnosed individuals are fairly reminiscent of those ‘good outcome’ individuals first diagnosed in childhood. Studies from adult ASD clinics suggest up to 50% are employed or in higher education (Happé et al., 2016, Hofvander et al., 2009) and 33–50% live independently (Hofvander et al., 2009, Lehnhardt et al., 2016). Ascertaining whether a behaviour-cognition mismatch truly characterised these individuals in earlier life, i.e. that ‘neurotypical’ behaviour masked core autistic difficulties, is challenging as there are no studies to our knowledge that address, even retrospectively, what these adults looked like as children. However, anecdotal and qualitative reports from these individuals themselves suggest that underlying difficulties were present long before the time of diagnosis; for example, in expressions of having felt different or misunderstood throughout their lives (e.g., Bargiela et al., 2016, Hickey et al., 2017). Accurately quantifying compensation in this late diagnosed group will also require sensitive theory of mind tasks, where task performance cannot rely on the very strategies that these individuals may have developed to ‘cope’ without a diagnosis up until adulthood.

### 3.4 Female autism presentation

Although ASD has historically been a predominantly male condition (Fombonne, 2009), there is mounting evidence to suggest that ASD may be more prevalent in females than previously assumed (Lai et al., 2015, Robinson et al., 2013). Females in general get diagnosed significantly later than their male counterparts (Begeer et al., 2013, Rutherford et al., 2016) and are more likely to receive an alternative diagnosis before ASD is confirmed (Bargiela et al., 2016, Begeer et al., 2013). One potential explanation (of several, see Lai et al., 2016) for later diagnosis amongst females is that females with high autistic traits are less likely to show clinically impairing symptoms due to a superior ability to compensate (Dworzynski et al., 2012). Support for this notion comes from cross-sectional observations that ASD symptom severity is equivalent between males and females in childhood, yet by adulthood, females display fewer social symptoms (Lai et al., 2011, Rynkiewicz et al., 2016).

Clinical observations and self-reports also suggest that females may be particularly motivated and/or skilled in ‘camouflaging’ their social difficulties (Mandy and Tchanturia, 2015, Simone, 2010), although these difficulties may continue to be experienced as impairing. Of interest here, a recent qualitative investigation (Hull et al., 2017) into ‘camouflaging’ in autistic adults distinguishes masking and compensation as two sub-components of camouflaging; the former reflecting suppression of autistic behaviours and the latter reflecting active strategies to support appearing ‘neurotypical’. Contrastingly, the framework presented in this paper would encompass both of these instances as compensation as they both fit our definition of a discrepancy between behaviour and underlying cognitive ability. Instead, we distinguish between simpler and fairly inflexible methods (e.g., suppressing a behaviour) with more sophisticated ones (e.g., explicitly developed strategies to reason about mental states), in terms of the depth of compensation (See Section 4.1).

Claims for advanced compensation in the female versus male autism phenotype would be best supported by evidence for less severe behavioural presentation in females versus males, despite equally severe ASD-related cognitive and/or brain atypicalities. Additionally, if this male-female disparity was found to be more distinct in older individuals, this would imply enhanced compensation in females developing across the lifetime. There has to our knowledge been only one preliminary investigation of how the discrepancy between observed behaviour and underlying cognition might differ by sex. Lai et al. (2016) explored the difference between standardised scores of ‘internal’ versus ‘external’ measures; theory of mind ability (inferred from performance on the Reading the Mind in the Eyes task; Baron-Cohen et al., 2001a) and self-rated Autism-Spectrum Quotient (AQ; Baron-Cohen et al., 2001b) scores, versus observer-rated symptoms (Autism Diagnostic Observation Schedule [ADOS]; Lord et al., 2000). They concluded that this difference, which they refer to as an ‘operationalised camouflaging measure’, was greater for diagnosed females than males. Further, a greater difference between internal and external levels, i.e., a greater ‘camouflaging’ score, was associated with medial-temporal based volume differences in the female brain only. The data showed that there was no sex difference on the Reading the Mind in the Eyes task and instead, the male-female difference in ‘camouflaging’ scores was driven by higher self-rated symptoms in females on the AQ, and higher observer-rated symptoms in males on the ADOS. This supports the notion that autistic females might appear less behaviourally severe than males, despite equally severe doses of cognitive deficit. We do, however, draw attention to a potential issue with the use of the AQ as a proxy of underlying cognitive difficulties, as higher AQ scores in autistic females versus males may simply reflect greater *awareness* rather than greater experience of underlying difficulties.

Studying the female versus male autism phenotype may be a useful way to gain insight into the mechanisms involved in compensation. So far, research has been limited (e.g., Lai et al., 2016) by the fact that females in the clinic are more likely to have accompanying intellectual and/or behavioural difficulties (Lai et al., 2015, van Wijngaarden-Cremers et al., 2014). Furthermore, our diagnostic tools, derived primarily from the study of autistic males, may be less sensitive to the female autism phenotype (Rutter et al., 2003), thereby increasing the chances of seriously under-estimating females’ behavioural impairments. There is therefore a limited amount that could be discovered about enhanced compensation in females by studying diagnosed individuals, who may be less likely to have compensatory resources due to additional impairments. The most informative approach to identifying successful female compensators may be through population-based studies (e.g., Brunsdon et al., 2015) of females who report high autistic traits and demonstrate poor socio-cognitive abilities, but nevertheless compensate sufficiently for these difficulties so that they sit below the diagnostic threshold for ASD. It is also possible that internalising problems experienced by autistic females may overshadow their autistic symptoms in a clinical assessment, thus delaying the time it takes to gain a diagnosis. This is particularly problematic if internalising symptoms have arisen from the effort of deliberately compensating in the first place.

## 4 Key characteristics of compensation

Consideration of the ASD literature has isolated four ASD-related phenomena, as described in Section 3, which fit our working definition of compensation. This process has simultaneously highlighted other potentially important characteristics of compensation that may contribute to theory development. Here, we propose three hypothetical features of compensation, with the aim of being able to unite fairly discrete examples of compensation (good outcome, at-risk individuals transitioning away from the autism phenotype, late diagnosis and female autism presentation) under one umbrella term, not only within one specific neurodevelopmental disorder group, such as ASD, but also in a transdiagnostic manner.

### 4.1 Compensation may be shallow or deep

Compensation is unlikely to be spread evenly, meaning that there will be certain difficulties more easily compensated for than others (Ullman and Pullman, 2015). For instance, in the case of ASD, learning to laugh at a joke that relies on mental state understanding when others do is much simpler than working out why the joke was funny in order to formulate an appropriate social response. It is therefore conceivable that the compensation sitting beneath the surface of ‘typical’ behaviour could either be fairly shallow or instead could extend much deeper (see Table 1). We suggest that ‘shallow compensation’ is akin to the use of a white stick by the visually impaired. It enables one to avoid obstacles, but does not go further in mimicking vision. By contrast, we suggest that echolocation would be ‘deep compensation’ as it allows the formation of a rich spatial representation (the end point of vision), albeit via a different route.

In ASD, examples of both shallow and deep compensation can be identified. In terms of the former, there are strategies that allow one to navigate the social world superficially (e.g., making deliberate eye contact, imitating others, inhibiting undesirable social behaviours). These strategies are inflexible, do not work well in novel situations or when social cues are especially ambiguous. Importantly, these strategies do not support underlying socio-cognitive processes, i.e., they would be unlikely to support intact performance on sensitive measures of theory of mind. Moreover, we predict that shallow compensation is fragile and can rapidly break down, particularly when compensatory resources are limited, such as under stress, anxiety or mental fatigue.

Shallow compensation might explain why even those autistic individuals with ‘good outcome’, who score well on assessments of autistic behaviours, often say they still struggle with day-to-day social scenarios. The Autism Diagnostic Observation Schedule (ADOS; Lord et al., 2000), a standardised one-to-one assessment, may in fact provide an optimal environment for compensation, since it involves structured interaction with a trained researcher in a quiet space. However, across multiple real-life social scenarios, the most difficult aspect to compensate for, i.e., a fundamental difficulty with intuitively understanding other minds, will continue to present challenges for many autistic individuals with so-called ‘good outcome’, as demonstrated in persistent difficulties with social relationships and meeting new people. Shallow compensation may also partially explain why those receiving a late diagnosis sometimes describe having ‘burnt out’, as shallow compensatory strategies become inefficient to support day-to-day social functioning in adulthood.

The possibility does remain that for certain autistic individuals, compensation extends deeper. That is to say that genuinely sophisticated alternative routes to good theory of mind performance might exist. These routes are unlikely to use the usual computational machinery that allows effortless representation of mental states in typically developing individuals from the second year of life, but may still provide a sufficient, albeit slower, route to mental state attribution. For instance, autistic individuals may rely on particular cognitive strengths, such as detail-focused perception and exact memory (see Happé and Frith, 2006), to process and analyse social information, although no empirical research has been conducted on this to date. We predict that deep compensation should be relatively more flexible and resistant to break down under stress and/or fatigue than shallow compensation.

Quantifying shallow compensation will require accurate measurement of both autistic behaviour and underlying social cognition and perhaps, exploration of how the severity of symptoms alters with the changing demands of the environment. Ideally, numerous measures of autistic behaviours across different contexts should be taken into account. We might expect shallow-compensated individuals to show a particularly uneven behavioural profile across multiple contexts (e.g., research setting, home/work setting, novel scenarios). If shallow compensation exists, then we should observe break down in certain contexts, when demands exceed compensatory ability. Quantifying *deep* compensation, however, will prove particularly challenging, especially if the supporting processes are sophisticated enough to support mental state attribution, even in novel scenarios or relatively difficult theory of mind tasks. Instead, investigating the underlying neural signatures (e.g., using fMRI or EEG) associated with good task performance might prove particularly useful by shedding light on the nature of the alternative route to good task performance.

### 4.2 Compensation is modulated by the environment

The immediate environment may serve either to facilitate or impede the workings of compensation. That is to say that the environment might directly modulate the extent to which cognitive difficulties are manifest in behaviour. This could be regarded as a form of external compensation (derived from external sources rather than those internal to the individual), which we here define as *environmental scaffolding*. For example, in the case of developmental dyslexia, the orthography you have to acquire has a major effect on the visibility of the phonological processing difficulties. In languages with regular phoneme-grapheme pairings (like Italian), few children are slow to learn to read, although dyslexics can still be identified using specific cognitive tasks (e.g., rapid picture naming). In the case of ASD, we can speculate that environments where social rules are very explicit will make it easier for an autistic person to know how to behave socially, thereby facilitating compensation. Equally, the environment may serve to impede or disrupt compensation; for example, increasing demands in the environment may outstrip compensatory capacity at particular points in development, thereby revealing autistic traits in behaviour. The child who had a predictable home environment tailored to their needs might be increasingly required to interact with others at school and into higher education and/or work. For those seeking diagnosis in adulthood, the task of compensating may have been achievable in childhood, up until a critical point when environmental demands (on e.g., social independence, self-care) increased substantially.

The potential for the environment to modulate compensatory processes and thereby, the expression of symptoms, should be distinguished from the ability of the environment to promote ‘good outcome’ for the individual, without the behavioural manifestation of symptoms necessarily lessening. We refer to this phenomenon as *environmental accommodation*. This may be particularly apparent as individuals begin to self-select their environments across development (e.g., particular academic and/or occupational settings) in order to match their abilities (e.g., strengths in detail processing, difficulties in social interaction), a phenomenon that Johnson et al. (2015b) have referred to as ‘niche construction’. Certain individuals may increasingly select environments that hold non-social abilities (i.e., autistic strengths) to a higher standard than social skills (i.e., autistic difficulties). Therefore, although these individuals may continue to display typically autistic behaviours, their immediate environment or niche is much more accommodating of these (or actually positively embraces these), such that their subjective quality of life and mental wellbeing are less likely to be negatively affected. There may be particular societies/cultures where environmental accommodation is more likely to take place. For example, in an environment or society where an individual does not have to read many other minds, there would be fewer opportunities for that individual’s poor theory of mind ability to have a detrimental effect on their quality of life. Future research should aim to highlight the different ways in which particular environments may i) directly interact with or modulate compensation, as well as ii) promote good outcome, independent of compensation.

### 4.3 Compensation may come at a cost

The very idea of compensation implies that a secondary, perhaps less well-suited set of cognitive resources/machinery is being used in the absence of resources/machinery that would typically serve the purpose. For example, domain-general resources may be used in the absence of domain-specific (e.g., socio-cognitive) ones. If these domain-general resources are finite (and shared with other tasks), they will rapidly exhaust after continuous use for compensation and equally, if they are preferentially allocated to compensation, this will be at the expense of other tasks dependent upon these domain-general resources. This hypothesis is in line with reports from autistic individuals themselves indicating that the task of ‘pretending to be normal’ during social interaction is mentally tiring and stressful (Bargiela et al., 2016; Hull et al., 2017). One young man with ASD, Russell Lehmann, describes the improvements in his own autistic symptoms from childhood to adulthood as “stemming from nothing but grueling, demanding, exhausting work” (personal communication).

The incidence of mental health problems amongst ‘compensated’ individuals might speak to the demanding and taxing nature of compensation. For instance, additional mental health difficulties amongst late diagnosed adults are the rule rather than the exception (Happé et al., 2016, Lever and Geurts, 2016b). Geurts and Jansen (2012) found that 53% of individuals coming for first diagnosis in adulthood and receiving an ASD diagnosis had previous contact with a mental health clinic and exhibited high levels of depression and anxiety symptoms. Additionally, Cassidy et al. (2014) reported extremely high prevalence of suicidal ideation (66%) amongst individuals attending an adult ASD clinic, which substantially exceeds estimated figures amongst adults diagnosed in childhood or adolescence (e.g., Balfe and Tanta, 2010). There are a number of possible explanations for this association between late diagnosis and heightened mental health problems. First, the effort of compensating across the lifetime might have a downstream detrimental impact on mental health. Second, those individuals who are more likely to compensate in the first place might be those with additional mental health problems; for example, a more anxious individual might be especially driven to ‘appear normal’. Lastly, but equally possible, additional mental health difficulties may have over-shadowed the presentation of autistic symptoms to the extent that individuals do not receive a diagnosis of ASD until after a lifetime of mis/partial diagnoses.

Interestingly, additional mental health difficulties (e.g., anxiety) are frequently reported amongst unaffected siblings of those with ASD (Hallett et al., 2013; Shephard et al., 2017). Whether these heightened mental health problems reflect a core characteristic of the BAP, an aetiological link between ASD and other psychiatric problems, the stressful experience of growing up with a sibling with ASD or instead, something important about the cost of compensation to those who transition away from the autism phenotype early in life, is an interesting avenue for future research. Finally, it can be noted that residual psychiatric difficulties have been reported even amongst those ‘optimal outcome’ individuals who no longer meet diagnostic criteria for ASD (Fein et al., 2013, Mukaddes et al., 2017).

The exact nature of the relationship between compensation and mental health difficulties, such as anxiety, depression and suicidal ideation, requires empirical clarification and in particular, a longitudinal approach. Importantly, distinguishing between shallow and deep compensation should help to further our understanding of the specific types of compensation, if any, that genuinely come at a cost to the individual. For example, there is reason to suspect that those individuals engaging in deep compensation, which permits relatively flexible social understanding, might not necessarily be vulnerable to subsequent mental health problems. This may particularly be the case when individuals seek niche environments for themselves that complement their array of cognitive abilities/deficits (i.e., when environmental accomodation takes place; see Section 4.2).

## 5 Potential neurocognitive mechanisms

The neurocognitive means by which compensation can be achieved is relatively unexplored. However, investigating those cognitive factors that have previously been associated with improved prognosis, i.e. ‘good outcome’, might be the most promising line of investigation. Additionally, we would expect the ability to compensate to be independent from the core cognitive burden for the disorder itself (Morton and Frith, 1994), if we are to account for the wide heterogeneity in the degree to which behavioural symptoms either persist or improve. This is corroborated by studies from the literature on ASD demonstrating that the severity of autistic symptoms in childhood is a poor predictor of the extent to which behavioural improvements will be made (Fountain et al., 2012, Levy and Perry, 2011). Instead, it should be informative to explore those cognitive factors that show a substantial degree of variance amongst individuals within the disorder group, yet remain intact for a proportion of individuals. It is possible that the compensatory role of these cognitive factors could be relevant transdiagnostically (see Ullman and Pullman, 2015) and we therefore include mention of other neurodevelopmental disorders in the examples below.

### 5.1 Intellectual ability

Neurodevelopmental disorders such as ASD are not defined by a particular level of intellectual ability, spanning the whole IQ range, from profound intellectual disability (IQ < 50) to average or above average intelligence (IQ > 115; Charman et al., 2011). ADHD is also suggested to be aetiologically distinct from intellectual disability, such that diagnoses can be seen amongst populations with low, average or high IQ (Katusic et al., 2011, Wood et al., 2011). In spite of the fact that IQ and severity of symptoms are not intrinsically linked (i.e., a severe symptom profile can exist alongside extremely high IQ), IQ might play a crucial role in the developmental unfolding of these disorders across the lifetime, i.e., changes in symptoms. Indeed, higher IQ in childhood is one of the strongest predictors of ‘good outcome’ in later life for autistic individuals (Billstedt et al., 2005, Blumberg et al., 2016, Howlin et al., 2004, Farley et al., 2009, Fein et al., 2013, Magiati et al., 2014). Interestingly, higher childhood IQ also characterises those that remit from ADHD symptoms by young adulthood, compared to those who persist (Cheung et al., 2016). IQ, therefore, may represent a key feature of the propensity/ability to compensate. Indeed, in ASD, there is evidence to suggest that greater IQ, in particular verbal IQ (Durrleman and Franck, 2015, Fisher et al., 2005, Happé, 1995), may help certain individuals to bootstrap their limited theory of mind ability. The exact nature of the relationship between IQ and compensation, however, requires clarification. It is equally possible that the propensity to compensate early in life might drive IQ throughout development (Anderson, 2008). For example, some early forms of compensation might facilitate social development, thereby providing an improved learning environment for acquiring the domain-general skills that are tapped in an IQ test. Finally, whether high IQ could support compensation across all settings is unknown. For example, IQ might best facilitate compensation in a shallow manner, within structured and predictable settings (e.g., an ADOS assessment). It may or may not be sufficient to support deeper compensation, in the fast-paced and unpredictable social settings of everyday life.

Good intellectual ability is also implicated in the previously discussed examples of compensation in ASD. For example, an fMRI study investigating neural correlates during a task of social exclusion in autistic adolescents, their unaffected siblings and typical controls, found that in the unaffected siblings only, higher IQ was associated with a more ‘typical’ neural activation pattern (Bolling et al., 2015). The authors suggest that in the face of substantial risk for ASD, higher IQ might steer certain siblings towards typical social cognition. In terms of the female autism phenotype, evidence suggests that those who do meet diagnostic criteria are more likely to have poorer IQ, which tips them over the diagnostic threshold, compared to other females with equally high autistic traits (Dworzynski et al., 2012). Whether females are simply less likely to come to clinical attention in the absence of additional intellectual difficulties due to the insensitivity of our diagnostic instruments, or because higher IQ genuinely plays a specific role in compensating for underlying difficulties is a crucial distinction to be made by future research (e.g., by examining the impact on mental health and quality of life).

Higher IQ also appears to be an important feature of the late diagnosed population. For example, Lehnhardt et al.’s (2016) study found that males and females diagnosed in adulthood had exceptionally high verbal and non-verbal IQ. Further, females had a significantly greater processing speed compared to males, as reflected in the Digit-Symbol-Coding subtest of the WAIS IQ test, which the authors interpreted as a particular compensatory advantage for females when processing and analysing fast social cues. Finally, cross-sectional studies of adults seeking a diagnosis in adulthood suggest that increasing age is associated with greater intellectual ability (Happé et al., 2016) and visual memory (Lever and Geurts, 2016b). One potential explanation of these findings is that older adults who have compensated for longer in their lifetime until seeking clinical support may have done so with the aid of enhanced intellectual abilities.

### 5.2 Executive function

Executive function refers to a constellation of higher-order cognitive abilities in planning, inhibition and cognitive flexibility, which may be fractionable from one another (Joseph and Tager-Flusberg, 2004). Although executive dysfunction has previously been proposed to play an aetiological role in the development of autistic symptoms (Ozonoff et al., 1991, Russell, 1997), particularly non-social symptoms (e.g. Yerys et al., 2009), there is increasing evidence to suggest that it is not a universal feature of ASD (Brunsdon et al., 2015, Cantio et al., 2016, Geurts et al., 2014, Wallace et al., 2016). This has led to the suggestion that executive dysfunction should be conceived as an individual difference amongst the autistic population, rather than as a core feature of ASD itself (Johnson, 2012). Instead, executive function might remain relatively intact for a proportion of autistic individuals and can therefore be recruited to support compensation. Indeed, Johnson et al. (2015b) speculate that those with a double hit of ASD and executive dysfunction might be completely stripped of compensatory resources and thus will be the most likely to exhibit a severe behavioural profile.

One can speculate about the mechanisms by which executive function ability might facilitate compensation, particularly shallow compensation. For example, greater ability to inhibit undesirable social behaviours, to plan behaviours before and throughout social interaction, and to be flexible in potentially unpredictable social scenarios might help to reduce observable social symptoms. There is also evidence to suggest that intact executive function might be important to those subgroups of individuals showing apparently good compensation. For example, Troyb et al. (2014) have reported comparable executive function ability across a range of tasks measuring inhibition, set-shifting, planning and working memory between ‘optimal outcome’ and typically developing individuals. Additionally, Lehnhardt et al. (2016) found that late diagnosed females had significantly greater executive function ability on tasks involving cognitive flexibility and processing speed, compared with late diagnosed males, suggesting that females who have ‘coped’ without a diagnosis until adulthood may have done so because of their good ability to monitor and regulate their social behaviour. Future exploration of how executive function and/or intellectual abilities might differ between early and late diagnosed groups will serve to clarify the degree to which these cognitive abilities do indeed specifically facilitate compensation amongst females and those at risk of late diagnosis. Moreover, the extent to which executive function plays a specific role in compensation over and above general intellectual ability remains to be seen. One study (Pugliese et al., 2015) suggests that executive function ability is a stronger predictor of ‘good outcome’ (in terms of better adaptive living skills) for autistic children and young adults, compared to general intelligence.

### 5.3 Neural mechanisms

Investigation at the neural level might be informative in determining the extent to which good behavioural, and possibly cognitive, performance is underpinned by engagement of the systems used by typically developing individuals, suggesting genuine remediation or delayed maturation (see Table 1), or instead, alternative pathways, suggesting compensation. There are numerous ways in which compensatory processing might be reflected in an atypical neural signature. First, neural compensation may be evident in extra ‘neural effort’ required from the same neural network used by neurotypicals. For example, in the ASD literature, there are examples of fMRI studies demonstrating hyper-activation of the so-called ‘theory of mind’ network (medial prefrontal cortex, posterior cingulate and lateral temporal cortices) when performing theory of mind tasks, implying that ‘social’ tasks are still solved via social means, albeit, atypically. For example, White et al. (2014) found that even autistic adolescents who consistently passed a battery of theory of mind tasks (i.e., exhibited ‘typical’ cognitive performance) demonstrated an atypical pattern of activation of the ‘theory of mind’ network similar to those individuals who consistently performed poorly. This latter finding highlights the strength of combining cognitive and neuroimaging methods when investigating compensation, in particular deep compensation, where cognitive task performance might remain intact.

Neural compensation may also be reflected in the recruitment of alternative networks that either replace or support the functioning of the dysfunctional network. Whether an alternative system could ever fully substitute for the functioning of the ‘theory of mind’ system is yet to be established. There is, however, evidence to support Johnson et al.’s (2015b) proposal that the PFC is a candidate brain region for compensatory processing, given its fundamental role in top-down coordination of other cortical regions. For instance, Kaiser et al. (2010) used fMRI whilst individuals with ASD, their unaffected siblings and controls, watched biological motion. Of particular interest, they found a unique pattern of activity, only for the unaffected siblings, in the superior temporal sulcus and most intriguingly, the PFC, suggesting that PFC-dependent brain activity might be characteristic of individuals at risk of ASD, who bypass an ASD trajectory via compensation. Evidence for recruitment of additional brain areas not otherwise used by typically developing individuals is also found in the literature on ADHD (for a review, see Fassbender and Schweitzer, 2009) and developmental dyslexia (see Section 2.3).

The alternative possibility is that, at a neural level, compensation will be represented rather idiosyncratically, such that there is great variability from one ‘compensated’ individual to another, reflecting the numerous possible pathways to compensation. Interestingly, evidence for this may be disorder-specific. For example, in the case of ASD, studies measuring neural activation during theory of mind tasks consistently find either hyper-activation (see references above) or hypo-activation (Kana et al., 2015, Lombardo et al., 2011, O’Nions et al., 2014) of the ‘theory of mind’ network, in both children and adults with ASD, rather than any evidence of the idiosyncratic neural patterns that might have been predicted *a priori* (Happé and Frith, 2014). Finally, it should be noted that compensation could be reflected in suppressed brain activity, as suggested in the aging literature (see Grady, 2012).

It remains to be investigated by future research which of the potential neural mechanisms outlined above would be the most successful form of compensatory processing. That is to say, which would be the most efficient and effective for supporting, for example, ‘typical’ social behaviour in an ADOS assessment (i.e. shallow compensation), and which would extend to supporting theory of mind task performance and everyday, flexible social interaction (i.e. deep compensation). We might suspect that those individuals engaging in shallow compensation will continue to exhibit persistent brain atypicalities that are characteristic of ASD. However, the possibility remains that those engaging in deep compensation may in fact demonstrate fewer ASD-relevant brain atypicalities compared to other autistic individuals, particularly with increasing age, as compensatory strategies are refined.

## 6 Implications of a compensation framework

Thinking about neurodevelopmental disorders within a compensation framework could have a range of implications for research and clinical practice. First, compensation might begin to explain why children who appear behaviourally similar in childhood (e.g., Fountain et al., 2012) can follow divergent pathways to outcome, thus demonstrating great heterogeneity in the degree to which their symptoms improve, persist or worsen. Second, evidence so far suggests that compensation might play a role in under- or mis-diagnosis in the clinic. Diagnostic procedures for neurodevelopmental disorders, such as ASD, continue to rely on the severity of behavioural symptoms presented to the clinical observer. And yet, if there exists a substantial behaviour-cognition mismatch for certain subgroups, including some females and late diagnosed adults, the extent of an individual’s underlying socio-cognitive difficulties would not necessarily be apparent in the clinic. An individual with very limited understanding of others’ minds may on the surface appear not sufficiently ‘impaired’ to warrant diagnosis, especially if compensation has supported not only improved behavioural presentation but also some level of independence (e.g., educational or occupational success). Furthermore, even autistic individuals who eventually achieve ‘good outcome’ and perhaps no longer meet diagnostic criteria for ASD, but endure persistent difficulties at the cognitive level, will continue to have important support needs that may otherwise be overlooked. Finally, those individuals that compensate sufficiently to sit just below the diagnostic threshold, but experience socio-cognitive difficulties comparable to other diagnosed individuals, not only risk mis-diagnosis (Rutter, 2011) but may also be the most vulnerable to mental health problems.

Ideally, joining cognitive tasks (e.g., theory of mind tasks) and biological markers (e.g., genetic and neural markers for ASD) with the assessment of behaviour in the clinic would be the optimal way to determine if individuals are compensating and as such, avoid under- or mis-diagnosis. However, at present, this is far from straightforward. The precise cognitive features that underpin conditions such as ASD are not unequivocally understood. In this paper, for the purpose of illustration, we have discussed theory of mind impairment as it is a widely reported core deficit in ASD (Happé, 2015), but there is an array of potential cognitive (Brunsdon and Happé, 2014) and socio-cognitive (Happé et al., 2017) atypicalities associated with ASD. Whilst we await improved characterisation of these cognitive deficits, as well as potential biological markers, ascertaining compensation in the clinic may be best done through observation of behaviour across multiple contexts that differ in social demands.

The study of compensation may also provide insight into supporting those on the autism spectrum. For example, we may hope to boost compensatory resources early on in individuals at risk for ASD (Johnson, 2012). Additionally, individuals that are limited in their compensatory ability, for instance, those with both low IQ and/or poor executive function, may represent a particularly vulnerable subgroup of individuals. And yet, we have here challenged the notion that compensation is a universally desirable process. It may be that those subgroups suspected to be engaging in good compensation, particularly shallow compensation, are also at risk for mental health problems. Therefore, one should perhaps be cautious in universally promoting the use of compensatory mechanisms as a means of fostering genuinely ‘good’ outcome amongst autistic individuals. Indeed, there is evidence to suggest that our objective, neurotypical definitions of ‘good outcome’, which dominate the literature, do not necessarily coincide with autistic individual’s self-rated quality of life (van Heijst and Geurts, 2015). More research is needed to identify neurodiverse concepts of ‘good outcome’, and thinking about neurodevelopmental disorders through a compensation lens may be helpful in this.

## 7 Limitations and outstanding questions

The impetus for defining and subsequently describing compensation in this paper was to synthesise the limited evidence so far for this phenomenon. For the purpose of demonstration, we have focused on one single cognitive deficit (theory of mind) within one condition (ASD), in order to explore how compensation might operate at behavioural and cognitive levels. In reality, there are likely multiple cognitive deficits that might be compensated for in different ways, across numerous conditions. This latter point feeds into the issue of heterogeneity, which is a defining feature of ASD. There may truly exist sub-groups of individuals who i) genuinely remit from ASD and its cognitive characteristics (as suggested by Fein et al., 2013), or ii) follow a more benign trajectory of the condition, thereby promoting good outcome via some mechanism other than compensation (e.g., delayed maturation). Further research will be required to detect the subtle but qualitative differences between these possible subgroups and their aetiological bases/biomarkers. In Table 1 we have outlined hypothetical distinctions between compensation (both shallow and deep) and the other mechanisms that might promote improved outcome. Most importantly, alternative explanations to compensation will only be plausible if there is good evidence to suggest that stable underlying difficulties no longer exist, which, as outlined earlier, will require sensitive cognitive probes. Ultimately the most robust method to address this question will be a longitudinal developmental approach.

The notion of compensation also poses intriguing questions about mechanisms underlying various interventions for neurodevelopmental conditions. For example, we can ask whether such interventions are attempting to genuinely alleviate core difficulties or whether they are in fact trying to promote a layer of compensation, which supports the individual to reach a more neurotypical outcome. In the case of interventions aimed at improving social skills during childhood/adolescence, it seems that these may be targeting shallow compensation; for example, explicitly teaching individuals rules for how to make and maintain eye contact, initiate conversation and behave appropriately in particular social scenarios. This form of training is likely inflexible and there is evidence for failure to generalise (for a review, see Fletcher-Watson et al., 2014). Early intensive interventions, however, (e.g., Early Start Denver Model, Dawson et al., 2010; Focused Playtime Intervention, Kasari et al., 2014), where there is an intention to rebuild the developmental components thought to be altered in ASD, such as joint attention and imitation, could be seen as aiming to alleviate the core features of the condition. Investigating neural markers may be helpful in uncovering whether such interventions are achieving compensation or instead, are addressing the core underlying cognitive deficits. Research so far on ‘optimal outcome’ following early intensive Applied Behavioural Analysis or ABA, suggests that brain functionality amongst these individuals still more closely resembles that of autistic rather than neurotypical individuals (e.g., Eigsti et al., 2016), thus suggesting compensation rather than genuine remediation.

## 8 Conclusions

The concept of compensation in neurodevelopmental disorders has received strikingly little theoretical or empirical consideration. We aimed to derive a transdiagnostic working definition of compensation in neurodevelopmental disorders, drawing inferences from the literature on developmental dyslexia, in order to i) review evidence for compensation in ASD and ii) develop a primary theoretical framework to guide future investigation of compensation. We define compensation as *the processes contributing to improved behavioural presentation of a neurodevelopmental disorder, despite persisting core deficit(s) at cognitive and/or neurobiological levels.* From the ASD literature we highlighted four instances where compensation might be taking place: good outcome, at-risk individuals transitioning away from the autism phenotype, late diagnosis and female autism presentation. Interestingly, these examples of compensation are likely to be relevant to other neurodevelopmental phenotypes, in particular ADHD, where there is also evidence for heterogeneity in outcome (Agnew-Blais et al., 2016, Steinhausen, 2009), at-risk siblings buffering against ADHD presentation (Johnson et al., 2015a), first diagnosis/emergence in adulthood (Caye et al., 2016) and under-diagnosis in females (Quinn and Madhoo, 2014).

On considering compensation in the ASD literature, we have hypothesised about a number of additional characteristics of compensation that we hope will act as a useful spring board for future research into the phenomenon, both in ASD and other neurodevelopmental disorders: i) compensation may be shallow or deep, ii) compensation is modulated by the environment, and iii) compensation may come at a cost. Additionally, we have speculated about potential cognitive abilities (e.g., intellectual ability, executive function) and neural patterns that might drive or reflect compensatory mechanisms across development. Finally, we have considered the potential utility of reframing neurodevelopmental disorders from a compensation perspective in order to advance research and clinical knowledge concerning heterogeneity, mis- or under-diagnosis and the high prevalence of co-morbid mental health problems in neurodevelopmental disorders. Ultimately, we have provided a framework from which novel hypotheses about compensation in these conditions can be tested. We propose that compensation is best viewed as a mismatch between behaviour and cognition, that its measurement will require both accurate behavioural and cognitive probes and finally, that investigation at the neural level might begin to answer questions about some of the most sophisticated forms of compensation that are hidden at the cognitive level.
